# Tooth Loss and Uncontrolled Diabetes Among Korean Adults With Type 2 Diabetes: Insights From the Korea National Health and Nutrition Examination Survey (KNHANES) 2014–2018

**DOI:** 10.1002/cre2.70371

**Published:** 2026-05-17

**Authors:** Eun Sik Lee, Gyu Bae Lee, Yusun Her, Koh Eun Shin, Kyung‐Hwan Cho, Kyungdo Han, Nai‐Wen Chang, Youngjun Lee, Byoungduck Han, Yang‐Hyun Kim

**Affiliations:** ^1^ Department of Family Medicine, Korea University Anam Hospital Korea University College of Medicine Seoul Korea; ^2^ Department of Statistics and Actuarial Science Soongsil University Seoul Korea; ^3^ Department of AI‐Bio, Graduate School of AI Soongsil University Seoul Korea; ^4^ Department of Periodontics Texas A&M University College of Dentistry Dallas Texas USA; ^5^ Texas A&M University College of Dentistry Dallas Texas USA

**Keywords:** elderly, glycosylated hemoglobin, the Korean National Health and Nutrition Examination Survey, tooth loss, type 2 diabetes, women's health

## Abstract

**Objectives:**

The relationship between tooth loss and glycemic control status in diabetes is inconclusive.

**Material and Methods:**

Using data from the 2014–2018 Korean National Health and Nutrition Examination Survey (KNHANES), 2146 adults with diabetes aged ≥ 40 years were categorized by number of remaining teeth (0–19, 20–27, and ≥ 28). Uncontrolled diabetes and poorer glycemic control were defined as HbA1c ≥ 6.5% and ≥ 7.0%, respectively. Multivariable logistic regression models calculated the odds ratio (OR) for uncontrolled diabetes and poorer glycemic control according to the number of remaining teeth.

**Results:**

No significant association was observed in men. In women, fewer remaining teeth were associated with higher odds of uncontrolled diabetes (per 1‐tooth increase OR 0.961, 95% CI 0.937–0.986; *p* = 0.002), and compared with ≥ 28 teeth, OR were higher in the 0–19 teeth (OR 2.500, 95% CI 1.535–4.074) and the 20–27 teeth (OR 1.679, 95% CI 1.073–2.629) (all *p* = 0.002). Furthermore, among women, the associations persisted in both 40–59 years (per 1‐tooth increase OR 0.878, 95% CI 0.780–0.988; *p* = 0.030) and ≥ 60 years (per 1‐tooth increase OR 0.965, 95% CI 0.939–0.992; *p* = 0.011). Among women aged ≥ 60 years, those with 0–19 teeth had higher odds of uncontrolled diabetes (OR 1.994, 95% CI 1.041–3.818; *p* = 0.029) and poorer glycemic control (OR 2.498, 95% CI 1.321–4.723; *p* = 0.008) compared with those with ≥ 28 teeth.

**Conclusions:**

Fewer remaining teeth were associated with poorer glycemic control among women with diabetes, independent of periodontitis. This association was most pronounced in women aged ≥ 60 years. Large cohort studies are warranted to clarify causality and to evaluate the roles of periodontitis and dental caries in this relationship.

**Abbreviations**
BMI: body mass index
CI: confidence interval
FBG: fasting blood glucose
HbA1c: glycosylated hemoglobin
HDL‐C: high‐density lipoprotein‐cholesterol
KNHANES: the Korea National Health and Nutrition Examination Survey
LDL‐C: low‐density lipoprotein‐cholesterol
OR: odds ratio
Q: quartile
T2DM: type 2 diabetes
TG: triglycerides

## 1 Background

Diabetes mellitus is a chronic metabolic disease characterized by hyperglycemia resulting from defects in insulin secretion or insulin sensitivity. Hyperglycemia contributes to life‐threatening complications in the heart, blood vessels, eyes, kidneys, and peripheral nerves (World Health Organization 2016). Type 2 diabetes (T2DM), characterized by insulin resistance, accounts for 90%–95% of all diabetes (American Diabetes Association Professional Practice Committee 2024). The global prevalence of diabetes is rapidly increasing due to an aging population, urbanization, and sedentary lifestyle changes (Zimmet et al.^2001^). According to the World Health Organization, an estimated 422 million adults worldwide had diabetes in 2014, compared to 108 million in 1980, and the low‐ and middle‐income countries were especially vulnerable to the increasing prevalence of T2DM (World Health Organization 2016). In 2020, it was estimated that 1 out of 6 (16.7%) Korean adults aged 30‐years or older had diabetes. The situation was particularly dire in the adult population older than 65 years, in which 30.1% had diabetes (Bae et al. 2022). Less than 70% of those with diabetes were aware of their condition. Although 60.1% were being treated, and only 25% had it well‐controlled, defined as meeting the glycosylated hemoglobin (HbA1c) target < 6.5% (Bae et al. 2022).

The risk factors for diabetes in Koreans include being overweight (body mass index [BMI] ≥ 23 kg/m^2^) or obese (BMI ≥ 25 kg/m^2^), abdominal obesity (≥ 90 cm for male; ≥ 85 cm for female), family history; age, hypertension, dyslipidemia, insulin resistance (e.g., acanthosis nigricans, polycystic ovary syndrome), cardiovascular diseases (coronary artery disease, strokes), or medications (glucocorticoids, atypical antipsychotics) (Choi et al. 2023). Periodontal disease, one of the most common chronic inflammatory diseases, destroys the connective tissue surrounding the teeth, eventually leading to tooth loss (Armitage 1999). Dental caries and its sequelae are also major risk factors for tooth loss. Additionally, DM is closely linked to tooth loss. Ahmadinia et al. (2022) concluded in a meta‐analysis of cross‐sectional studies that diabetes is significantly associated with tooth loss. Accordingly, the American Diabetes Association has recognized the importance of screening for dental complications and comorbidities during the initial clinical encounter (American Diabetes Association Professional Practice Committee 2024).

Available evidence supports the robust relationship between tooth loss and poor control of diabetes in the vulnerable population. Taylor et al. (2004) highlighted that DM complications impose a heavier burden on minority population and economically disadvantaged groups in the United States were especially susceptible to periodontal diseases and tooth loss. Greenblatt et al. (2016) found a positive relationship between poor diabetic control and tooth loss in the Hispanic/Latino population. Yet, data are mixed on which age‐specific subgroups are more vulnerable (Ahmadinia et al. 2022; Kapp et al. 2007). The positive association between tooth loss and the severity of diabetes was previously noted in the Korean population (Yoo et al. 2019). However, no study looked into the relationship between tooth loss and the precise glycemic control status in specific subgroups of age and sex at the population scale. Therefore, we investigated the association between tooth loss and the degree of glycemic control using representative sample data of the Korean population.

## 2 Methods

### 2.1 Survey Overview

In this study, we included the data from the Korea National Health and Nutrition Examination Survey (KNHANES) from 2014 to 2018. The Korea Disease Control and Prevention Agency governs the KNHANES, which consists of the nutrition survey, health examination, and health interview. Every year, a total of 10,000 individuals aged over 1 year from 192 regions participate in the KNHANES. The health examination and interview were conducted in the mobile medical vehicles, and the nutrition survey was completed at home 1 week after the health interview. Trained professionals, including nurses, dietitians, and health scientists, conducted the face‐to‐face interviews using systematic questionnaires.

### 2.2 Study Population

A total of 30,633 participants were included in the 2014–2018 KNHANES. We excluded those < 40 years (*n* = 12,854) because prior KNHANES studies have excluded participants in their 20s and 30s to reduce the likelihood of including individuals with type 1 diabetes (Kim, Won et al. 2016; Kim et al. 2024). Among the remaining 17,779 participants, 892 did not fast for at least 8 h prior to the blood test; 14,347 did not have diabetes; 84 lacked information on the major variables (remaining teeth and HbA1c); and 310 had missing data (e.g., demographic information). The remaining 2146 adults, 1125 men and 1021 women, diagnosed with diabetes were included in this study. The study was conducted according to the Ethical Principles for Medical Research Involving Human Subjects as stated in the Helsinki Declaration. All subjects provided written informed consent.

### 2.3 Measurement of Remaining Teeth

The oral examination was performed by trained dental specialists. Tooth count was calculated excluding third molars (maximum 28 teeth) and categorized as 0–19 (below functional dentition), 20–27 (partial tooth loss), and ≥ 28 (complete dentition). This categorization reflects the WHO concept of functional dentition (≥ 20 teeth sufficient for adequate mastication) and follows classifications used in prior population‐based studies in Korea (Organization WH 2013; Kim, Cho et al. 2016; Hwang and Park 2024; Kim et al. 2023). In line with prior KNHANES analyses, third molars (wisdom teeth) were excluded when calculating the number of remaining teeth (Park et al. 2019; Cho et al. 2021). We analyzed 2146 participants with diabetes, including one edentulous participant, and categorized them by the number of remaining teeth (0–19, 20–27, or ≥ 28).

### 2.4 Periodontitis

Periodontal status was evaluated using the World Health Organization (WHO) Community Periodontal Index (CPI), assessed by trained examiners with the WHO CPI probe (Preshaw 2015). The oral cavity was divided into six sextants, and probing was performed on the designated index teeth (17, 16, 11, 26, 27, 36, 37, 31, 46, and 47) or on remaining teeth within the sextant when index teeth were missing. For each sextant, the highest CPI code observed was recorded, and CPI codes ranged from 0 to 4, representing healthy tissue (0), bleeding on probing (1), calculus (2), shallow periodontal pockets (3), and deep periodontal pockets (4). CPI code 3 indicated shallow pocketing (4–5 mm), whereas CPI code 4 indicated deep pocketing (≥ 6 mm) (Cho et al. 2021). For the present study, periodontitis was defined as having CPI code 3 or 4 in at least one sextant. In models examining the association between tooth loss and diabetes status, periodontitis was included as an adjustment covariate to account for underlying periodontal disease severity.

### 2.5 Definitions of Diabetes and Diabetic Control

We used American Diabetes Association (ADA), Korean Diabetes Association (KDA), and American Association of Clinical Endocrinology (AACE) guidelines to evaluate the glycemic status and glycemic control of participants (American Diabetes Association Professional Practice Committee 2024; Choi et al. 2023; Samson et al. 2023). Individuals with diabetes were defined as those with a fasting blood glucose (FBG) level ≥ 126 mg/dL, those taking any antidiabetic medication, including both oral hypoglycemic agents or insulin injection, or those with a physician's diagnosis of diabetes. Individuals with HbA1c level < 6.5% were defined as having well‐controlled diabetes, and those with HbA1c level ≥ 6.5% were defined as having uncontrolled diabetes in accordance with KDA and AACE guidelines (Choi et al. 2023; Samson et al. 2023). We also performed the secondary analysis using HbA1c level ≥ 7.0% as poorer glycemic control (Tables S1 and S2) because the ADA guideline defined HbA1c level < 7.0% as well‐controlled diabetes for non‐pregnant adults (American Diabetes Association Professional Practice Committee 2024).

### 2.6 Biochemical Measurements

We obtained blood samples from the participants after a minimum of 8 h fasting. Serum levels of FBG, HbA1c, total cholesterol, low‐density lipoprotein‐cholesterol (LDL‐C), high‐density lipoprotein‐cholesterol (HDL‐C), and triglycerides (TG) were enzymatically measured using a Hitachi Automatic Analyzer 7600 (Hitachi, Tokyo, Japan) at the Central Testing Institute in Seoul, Korea.

### 2.7 Socio‐Demographic and General Health Behaviors

The socio‐demographic and general health data from the participants were gathered using the self‐reported questionnaires. The questionnaires inquired about age, sex, smoking history, alcohol consumption, physical activity, education level, and household income. We defined current smokers as smokers at the time of the study and non‐smokers as those who had never smoked or who had smoked fewer than 100 cigarettes in their lifetime. The remainder were classified as ex‐smokers. We defined heavy drinkers as those who consumed more than three glasses of alcohol per day (30 g/day), and mild to moderate drinkers were those who consumed less than three glasses per day (15–30 g/day). Physical activity was evaluated using the International Physical Activity Questionnaire (Hagströmer et al. 2006). Exercising more than five times per week for 30 min/session was defined as regular exercise. Strenuous exercise more than three times a week for 20 min/session was also considered as regular exercise. We categorized education level as either high school graduate (≥ 13 years) or not. Household income was modified based on the number of household members and divided into quartiles, with Q1 being the lowest and Q4 being the highest.

### 2.8 Statistical Analysis

Data are provided as the mean ±standard error (SE) for continuous variables or as a percentage (%) for categorical variables. An unpaired *t*‐test was used for continuous variables, and the Chi‐square test was used for categorical variables. We utilized multivariable logistic regression analysis to calculate the odds ratio (OR) and 95% confidence interval (CI) for uncontrolled diabetes (HbA1c ≥ 6.5%) among the three remaining teeth categories after adjusting for covariates. Dyslipidemia was included as a covariate to reduce potential confounding, as it is associated with both tooth loss and diabetes control through shared metabolic and behavioral risk factors. A restricted cubic spline curve with four knots (df = 3) was utilized to evaluate if any non‐linearity existed between age and diabetes control (Figure S1). Because there was no significant non‐linearity between age and diabetes control (*p* for non‐linearity = 0.4626), age was modeled as a linear variable in Table 3 and Table S1. The SAS software package version 9.2 (SAS Institute, Cary, NC) was used for statistical analyses. All statistical tests were two‐tailed. We regarded the difference as statistically significant when the *p*‐value was less than 0.05.

## 3 Results

Table 1 shows the baseline characteristics of the total participants. Women were older and more frequently belonged to the lowest income quartile (all *p* < 0.001), and had a higher prevalence of hypertension and dyslipidemia than men (*p* = 0.033 and *p* < 0.001, respectively). In contrast, men were more likely to report heavy alcohol intake, current smoking, and physical activity, as well as having ≥ 13 years of education (all *p* < 0.001). There were no sex differences in remaining teeth (continuous or categorical), glycemic indices (FBG or HbA1c), or BMI, whereas periodontitis was more prevalent in men than in women (*p* < 0.001) (Table 1).

**Table 1:** General characteristics of study participants.

*n*	Total population	Men	Women	*p*‐value
	2146	1125	1021	
Age (years)	61.81 ± 0.31	60.36 ± 0.41	63.61 ± 0.41	< 0.001
Age (%, ≥ 60 years)	58.37 (1.40)	53.19 (1.95)	64.77 (1.90)	< 0.001
Remaining teeth (*n*)	22.05 ± 0.17	22.13 ± 0.23	21.95 ± 0.24	0.569
Weight (kg)	66.27 ± 0.32	71.17 ± 0.44	60.21 ± 0.33	< 0.001
Height (cm)	161.94 ± 0.26	168.18 ± 0.26	154.22 ± 0.22	< 0.001
Body mass index (kg/m^2^)	25.18 ± 0.09	25.10 ± 0.12	25.29 ± 0.13	0.272
Weight circumference (cm)	87.76 ± 0.23	89.32 ± 0.32	85.82 ± 0.33	< 0.001
Fasting blood glucose (mg/dL)	144.00 ± 1.06	145.50 ± 1.53	142.14 ± 1.50	0.125
HbA1c (%)	7.23 ± 0.04	7.26 ± 0.05	7.20 ± 0.05	0.449
Heavy alcohol intake (yes, %)	10.56 (0.88)	17.94 (1.53)	1.43 (0.41)	< 0.001
Current smoking (yes, %)	20.91 (1.18)	34.91 (1.92)	3.57 (0.71)	< 0.001
Physical activity (yes, %)	41.36 (1.32)	46.87 (1.88)	34.54 (1.76)	< 0.001
Education (%, ≥ 13 years)	19.93 (1.10)	28.42 (1.66)	9.43 (1.09)	< 0.001
Income (Q1) (yes, %)	29.64 (1.32)	24.25 (1.63)	36.31 (1.82)	< 0.001
Hypertension	59.57 (1.38)	57.03 (1.99)	62.73 (1.83)	0.033
Dyslipidemia	39.51 (1.35)	34.79 (1.84)	45.34 (1.93)	< 0.001
HbA1c (%)				0.312
< 6.5	29.71 (1.27)	30.84 (1.70)	28.32 (1.75)	
6.5–6.9	22.53 (1.06)	21.15 (1.51)	24.23 (1.48)	
≥ 7	47.76 (1.43)	48.02 (2.03)	47.45 (1.95)	
Remaining teeth groups (n)				0.411
< 20	26.18 (1.14)	25.98 (1.52)	26.42 (1.55)	
20–27	51.74 (1.45)	53.09 (1.94)	50.06 (1.94)	
≥ 28	22.09 (1.25)	20.92 (1.68)	23.52 (1.78)	
Periodontitis	53.40 (1.53)	59.21 (2.10)	46.21 (2.03)	< 0.001

*Note:* The *p*‐values were obtained using an unpaired *t*‐test or Chi‐square test.

Abbreviation: HbA1c, glycosylated hemoglobin.

**Figure 1:**
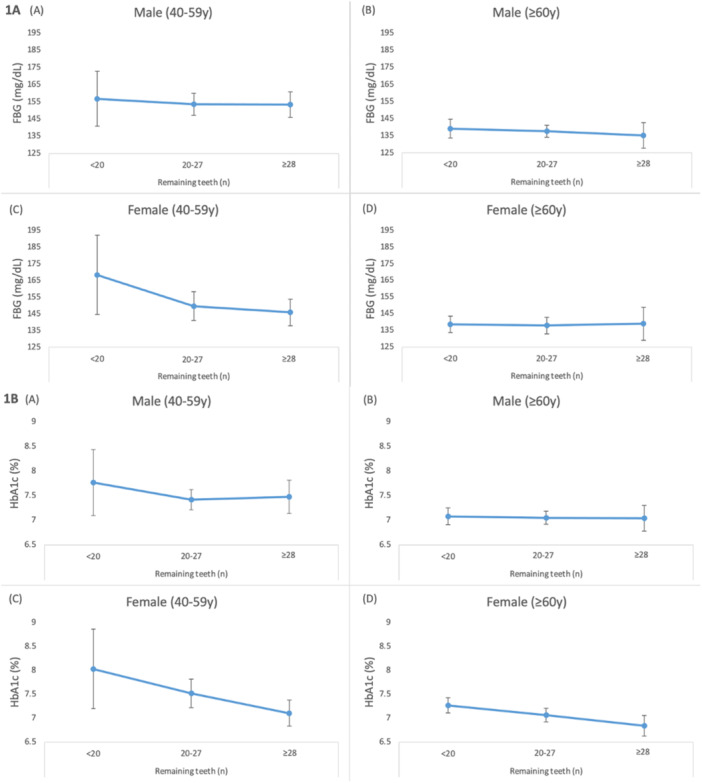
(1A): The average fasting blood glucose (FBG) values according to the number of remaining teeth in male and female stratified by age groups [(A) male 40–59 years, (B) male ≥ 60 years, (C) female 40–59 years, and (D) female ≥ 60 years]. (1B): The average glycosylated hemoglobin (HbA1c) values according to the number of remaining teeth in male and female stratified by age groups [(A) male 40–59 years, (B) male ≥ 60 years, (C) female 40–59 years, and (D) female ≥ 60 years].

Across both age groups (40–59 years and ≥ 60 years), mean FBG and HbA1c levels tended to increase as the number of remaining teeth decreased in both men and women, indicating a consistent trend toward poorer glycemic control with greater tooth loss (Figure 1A,B).

The number of remaining teeth according to HbA1c level in the total population and by sex and age group is shown in Table 2. In women, fewer remaining teeth were correlated with higher HbA1c levels (Table 2, Model 1, *p* = 0.036), and this inverse association remained significant after full adjustment for age, sex, BMI, smoking, drinking, exercise, education, income, hypertension, dyslipidemia, and periodontitis (Table 2, Model 2, *p* < 0.001).

**Table 2:** The number of remaining teeth according to HbA1c.

Remaining teeth (*n*)	Total population		Men		Women		Age 40–59 years		Age ≥ 60 years	
	Unweighted *n*	Mean ± SE	Unweighted *n*	Mean ± SE	Unweighted *n*	Mean ± SE	Unweighted *n*	Mean ± SE	Unweighted *n*	Mean ± SE
*Model 1*										
HbA1c < 6.5%	639	22.17 ± 0.31	356	21.70 ± 0.42	283	22.81 ± 0.41	195	25.40 ± 0.29	444	19.97 ± 0.39
6.5–6.9	504	21.88 ± 0.35	247	21.76 ± 0.48	257	22.01 ± 0.46	144	25.19 ± 0.41	360	20.30 ± 0.43
≥ 7	1003	22.05 ± 0.25	522	22.56 ± 0.34	481	21.41 ± 0.35	385	24.79 ± 0.28	618	19.66 ± 0.36
*p*‐value		0.827		0.178		0.036		0.302		0.510
*Model 2*										
HbA1c < 6.5%	639	21.89 ± 0.25	356	21.45 ± 0.35	283	22.44 ± 0.34	195	25.36 ± 0.29	444	20.12 ± 0.36
6.5–6.9	504	21.74 ± 0.31	247	21.53 ± 0.41	257	21.97 ± 0.44	144	25.19 ± 0.38	360	19.94 ± 0.41
≥ 7	1003	21.24 ± 0.23	522	21.66 ± 0.32	481	20.79 ± 0.31	385	24.84 ± 0.25	618	19.36 ± 0.34
*p*‐value		0.099		0.899		0.001		0.372		0.247
*Model 3*										
HbA1c < 6.5%	639	21.88 ± 0.25	356	21.45 ± 0.35	283	22.45 ± 0.33	195	25.38 ± 0.28	444	20.14 ± 0.36
6.5–6.9	504	21.77 ± 0.31	247	21.55 ± 0.41	257	22.00 ± 0.43	144	25.12 ± 0.38	360	19.97 ± 0.40
≥ 7	1003	21.22 ± 0.23	522	21.65 ± 0.32	481	20.75 ± 0.30	385	24.87 ± 0.25	618	19.31 ± 0.33
*p*‐value		0.080		0.91		0.001		0.388		0.18

*Note:* The *p*‐values were obtained using analysis of covariance (ANCOVA). Model 1 non‐adjusted. Model 2 was adjusted for age, sex, body mass index, smoking status, alcohol consumption, regular exercise, education level, household income, and presence of hypertension and dyslipidemia. Model 3 was adjusted for age, sex, body mass index, smoking status, alcohol consumption, regular exercise, education level, household income, and presence of hypertension, dyslipidemia, and periodontitis.

Abbreviation: HbA1c, glycosylated hemoglobin.

The relationship between the number of remaining teeth and uncontrolled DM according to the guidelines of KDA and AACE (HbA1c ≥ 6.5%) is shown in Table 3. The association differed significantly by sex for both continuous and categorical models (*p* for interaction 0.008 and 0.002, respectively). In men, no significant association was observed between remaining teeth and uncontrolled DM (*p* = 0.889). In women, fewer remaining teeth were associated with higher odds of uncontrolled DM when modeled continuously (per 1‐tooth increase: OR 0.961, 95% CI 0.937–0.986; *p* = 0.002). When categorized, the odds of uncontrolled DM were higher in the 0–19 teeth group (OR 2.500, 95% CI 1.535–4.074) and the 20–27 teeth group (OR 1.679, 95% CI 1.073–2.629) compared with the ≥ 28 teeth group (*p* = 0.002) (Table 3). Using a more stringent definition of uncontrolled DM (HbA1c ≥ 7.0%) yielded consistent findings (Table S1), with significant effect modification by sex in both the continuous and categorical models (*p* for interaction=0.002 and 0.007, respectively). In women, fewer remaining teeth were associated with higher odds of poorer glycemic control (per 1‐tooth increase: OR 0.956, 95% CI 0.934–0.979; *p* < 0.001), and the odds were higher in the 0–19 teeth group (OR 2.400, 95% CI 1.489–3.869) and the 20–27 teeth group (OR 1.634, 95% CI 1.060–2.519) compared with the ≥ 28 teeth group (*p* = 0.004).

**Table 3:** Adjusted odds ratios (95% CI) for uncontrolled DM (HbA1c ≥ 6.5%) according to the number of remaining teeth by sex.

Remaining teeth (*n*)	Total population			Sex						
				Men			Women			*p* for interaction
	Unweighted *n*	Event *n*	Adjusted OR (95% C.I)	Unweighted *n*	Event *n*	Adjusted OR (95% C.I)	Unweighted *n*	Event *n*	Adjusted OR (95% C.I)	
Per 1 tooth (continuous)	2146	1507	0.986 (0.969, 1.003)	1125	769	**1.005 (0.981, 1.029)**	1021	738	**0.961 (0.937, 0.986)**	**0.008**
*p*‐value			0.11			0.694			**0.002**	
0–19	615	432	1.424 (0.987, 2.054)	318	208	0.892 (0.525, 1.515)	297	224	**2.500 (1.535, 4.074)**	**0.002**
20–27	1121	794	1.208 (0.859, 1.697)	598	413	0.889 (0.554, 1.427)	523	381	**1.679 (1.073, 2.629)**	
≥ 28	410	281	1 (Ref.)	209	148	1 (Ref.)	201	133	1 (Ref.)	
*p*‐value			0.152			0.889			**0.002**	

*Note:* The *p*‐values were obtained using logistic regression. This model was adjusted for age, sex, body mass index, smoking status, alcohol consumption, regular exercise, education level, household income, and presence of hypertension, dyslipidemia, and periodontitis. Bold values indicate *p* values are < 0.005.

Abbreviation: OR, odds ratio.

Table 4 presents the association between the number of remaining teeth and uncontrolled DM (HbA1c ≥ 6.5%), stratified by sex and age. In men, no significant associations were observed in either 40–59 or ≥ 60 years stratum. In women, fewer remaining teeth were associated with higher odds of uncontrolled DM in both age strata when modeled continuously (age 40–59 years: per 1‐tooth increase OR 0.878, 95% CI 0.780–0.988; *p* = 0.030; age ≥ 60 years: OR 0.965, 95% CI 0.939–0.992; *p* = 0.011). When categorized, women aged ≥ 60 years displayed higher odds of uncontrolled DM in the 0–19 teeth group compared with the ≥ 28 teeth group (OR 1.994, 95% CI 1.041–3.818; *p* = 0.029), while women aged 40–59 years showed elevated odds in the 0–19 teeth (OR 4.615, 95% CI 1.091–19.525) and 20–27 teeth groups (OR 2.021, 95% CI 1.055–3.869) compared with ≥ 28 teeth, although the overall association was borderline (*p* = 0.054). Using a stricter definition of poorer glycemic control (HbA1c ≥ 7.0%), the association remained significant only among women aged ≥ 60 years (Table S2). In women aged ≥ 60 years, fewer remaining teeth were associated with higher odds of poorer glycemic control (per 1‐tooth increase: OR 0.957, 95% CI 0.933–0.981; *p* < 0.001), and women with 0–19 teeth had higher odds compared with those with ≥ 28 teeth (OR 2.498, 95% CI 1.321–4.723; *p* = 0.008).

**Table 4:** Adjusted odds ratios (95% CI) for uncontrolled DM (HbA1c ≥ 6.5%) according to the number of remaining teeth by sex and age group.

Remaining teeth (*n*)	Men					
	Age 40–59 years			Age ≥ 60 years		
	Unweighted *n*	Event *n*	Adjusted OR (95% C.I)	Unweighted *n*	Event *n*	Adjusted OR (95% C.I)
Per 1 tooth (continuous)	421	309	1.001 (0.947, 1.057)	704	460	1.005 (0.980, 1.031)
*p*‐value			0.984			0.698
0–19	54	37	0.902 (0.373, 2.180)	264	171	0.970 (0.505, 1.863)
20–27	231	172	0.790 (0.413, 1.512)	367	241	1.064 (0.578, 1.960)
≥ 28	136	100	1 (Ref.)	73	48	1 (Ref.)
*p*‐value			0.710			0.947
**Remaining teeth (*n*)**	Women					
	Age 40–59 years			Age ≥ 60 years		
	Unweighted *n*	Event *n*	Adjusted OR (95% C.I)	Unweighted *n*	Event *n*	Adjusted OR (95% C.I)
Per 1 tooth (continuous)	303	220	**0.878 (0.780, 0.988)**	718	518	**0.965 (0.939, 0.992)**
*p*‐value			**0.03**			**0.011**
0–19	22	19	**4.615 (1.091, 19.525)**	275	205	**1.994 (1.041, 3.818)**
20–27	161	121	**2.021 (1.055, 3.869)**	362	260	1.322 (0.701, 2.491)
≥ 28	120	80	1 (Ref.)	81	53	1 (Ref.)
*p*‐value			0.054			**0.029**

*Note:* The *p*‐values were obtained using logistic regression. This model was adjusted for age, sex, body mass index, smoking status, alcohol consumption, regular exercise, education level, household income, and presence of hypertension, dyslipidemia, and periodontitis. Bold values indicate *p* values are < 0.005.

Abbreviation: OR, odds ratio.

## 4 Discussion

In this nationwide study, women with < 20 teeth had 2.5‐fold higher odds of uncontrolled DM (HbA1c ≥ 6.5%) when compared with ≥ 28 teeth. Among women aged 40–59 years and those aged ≥ 60 years, having < 20 teeth showed 4.6‐fold and twofold higher odds of uncontrolled DM compared with women with complete dentition (≥ 28 teeth), respectively. Furthermore, among women aged ≥ 60 years, having < 20 teeth had 2.5‐fold higher odds of poorer glycemic control (HbA1c ≥ 7.0%) compared with complete dentition. In contrast, no association was observed between tooth loss and uncontrolled diabetes in Korean men, regardless of age.

The findings of the current study are broadly consistent with prior literature linking tooth loss to diabetes and glycemic status. Greenblatt et al. (2016) reported that Hispanic/Latino adults with uncontrolled diabetes were more likely to have substantial tooth loss than those with normal glycemic status. In Germany, Kaur et al. (2009) also demonstrated a positive association between type 2 diabetes and the extent of tooth loss among women. Similarly, our study showed that fewer remaining teeth were associated with poorer glycemic control, particularly in women. However, our results also extend previous studies. Even after adjusting for periodontitis, we demonstrated that the association between fewer remaining teeth and poor glycemic control persisted and that this pattern was most pronounced among women (Table 3 and Table S1).

Prior studies have emphasized periodontitis as a key pathway linking tooth loss to DM (Yoo et al. 2019; Yonekura et al. 2017). In the present study, by contrast, the association between tooth loss and poor glycemic control persisted even after adjusting for periodontitis, particularly in women, suggesting that factors beyond periodontitis may contribute to this relationship. Because tooth loss is commonly driven by both dental caries and its sequelae and periodontitis, our findings raise the possibility that periodontitis alone may not fully explain the link between fewer remaining teeth and poor glycemic control. Future studies should explore the impact of dental caries on the association between tooth loss and diabetes control.

The association between tooth loss and uncontrolled diabetes may be explained by the following mechanisms. Dental caries and periodontal disease are the two major causes of tooth loss (Jimenez et al. 2012; Fure and Zickert 1997). In cases of apical infection, bacteria and toxins, the source of dental caries and periodontal disease, can directly enter the bloodstream and cause bacteremia or systemic inflammation (Padilla et al. 2006). Also, the inflammatory mediators produced from the periodontal diseases can spread via the bloodstream and cause systemic inflammation (Taylor et al. 2013; Iacopino 2001). This systemic inflammation can induce proinflammatory cytokines such as C‐reactive protein, Interleukin‐1 (IL‐1), IL‐6, and tumor necrosis factor, potentially altering the action of insulin and causing impaired glycemic control and uncontrolled diabetes (Graves and Cochran 2003; Howells 1995). Rather than tooth loss directly causing uncontrolled diabetes, it is more likely that pro‐inflammatory cytokines and worsening insulin resistance drive tooth loss, which in turn contribute to poor glycemic control.

A sex‐based difference was observed in this study. This can be partly explained by the postmenopausal female population included in the study. Menopause, defined as a cessation of menstruation for at least 12 months, usually begins at age between late 40s and early 50s (Choe and Sung 2020). The level of estrogen begins to drop when women approach menopause. Many physical and psychological changes appear due to this hormonal change, and the most significant associated problem is osteoporosis (Güncü et al. 2005). Osteoporosis results in reduced bone mass, fragility, and an increased risk of fractures (Wactawski‐Wende et al. 1996). It also leads to a decrease in crestal alveolar bone density and a faster loss of bone height when triggered by factors like periodontal infection (Wactawski‐Wende et al. 1996). Impaired glycemic control from insulin resistance can provide this periodontal infection stimulus, causing more accelerated tooth loss in women compared to men.

This study had limitations. First, although the current study raised the possibility that periodontitis alone may not fully explain the link between tooth loss and poor glycemic control in women, given the cross‐sectional design, causal inference is limited. Future large cohort studies should evaluate the independent and joint contributions of periodontitis and dental caries to tooth loss and glycemic control. Second, the study was limited to the Korean population. Although a large representative sample of a homogenous ethnic group was analyzed, multiethnic data will allow for more generalizable results. Third, we lacked detailed dental history, including the duration or timing of tooth loss, dental caries status, and recent dental procedures, which may have contributed to residual confounding. In addition, periodontal measures such as clinical attachment loss (distance from the cemento‐enamel junction to the base of the periodontal pocket) and radiographic interdental bone loss were not available; inclusion of these measures could have further reduced potential confounding. Fourth, the patient's dietary habits, which could have influenced the glycemic control, were not included. Fifth, odds ratio estimates in women aged 40–59 years with ≤ 19 remaining teeth were based on small numbers of participants and events and therefore yielded wide confidence intervals (Table 4). These findings should be interpreted cautiously and warrant confirmation in future large cohort studies. Sixth, we were unable to study the impact of dental caries on the association between tooth loss and diabetes control. However, the present study suggests that periodontitis may not have much impact on the association between tooth loss and diabetes control in Korean adults. Despite these limitations, this study had numerous strengths. We used a representative sample of a homogenous ethnic population. This was the first population‐level study to examine the relationship between tooth loss and precise glycemic control status according to age and sex. Furthermore, the robust association between tooth loss and diabetes control persisted after adjusting for age, sex, body mass index, smoking status, alcohol consumption, regular exercise, education level, household income, and the presence of hypertension, dyslipidemia, and periodontitis.

## 5 Conclusions

Tooth loss was associated with poor diabetes control in Korean women aged 40 years and older. The greater the tooth loss, the worse the glycemic status was among the women. Particularly, women aged 60 years and older were most vulnerable to this association. This finding calls for a multidisciplinary approach, such as routine dental evaluations during diabetes care management. It may confer additional benefits in both glycemic and dental health in older women with diabetes. Large cohort studies are needed to verify the causal relationship between tooth loss and diabetic control and to evaluate the independent and joint contributions of periodontitis and dental caries to tooth loss and glycemic control.

## Author Contributions

Eun Sik Lee contributed to the conceptualization and writing of the original draft. Gyu Bae Lee contributed to conceptualization and writing – original draft, review and editing. Yusun Her, Koh Eun Shin, Kyung‐Hwan Cho, Nai‐Wen Chang, and Youngjun Lee contributed to writing – review and editing. Kyungdo Han contributed to data curation, formal analysis, and methodology. Byoungduck Han contributed to conceptualization and writing – review and editing. Yang‐Hyun Kim contributed to conceptualization, data curation, methodology, writing – original draft, and writing – review and editing. He is the guarantor of this work and, as such, had full access to all the data in the study and takes responsibility for the integrity of the data and the accuracy of the data analysis.

## Ethics Statement

The current study complies with the Declaration of Helsinki, and it was approved by the Institutional Review Board of Korea University Anam Hospital (IRB No. 2019AN0038). De‐identified and anonymized data were used for the analyses.

## Consent

The requirement for informed consent was waived for this study.

## Conflicts of Interest

The authors declare no conflicts of interest.

## Supporting information

Supporting File 1

Supporting File 2

## Data Availability

The data that support the findings of this study are available from the Korea National Health & Nutrition Examination Survey, https://knhanes.kdca.go.kr/knhanes/eng/main.do#. The data that support the findings of this study are available from the corresponding author upon reasonable request.
