# Supplementary material for: Tooth Loss and Uncontrolled Diabetes Among Korean Adults With Type 2 Diabetes: Insights From the Korea National Health and Nutrition Examination Survey (KNHANES) 2014–2018
Source: Clin Exp Dent Res. 2026 May 17;12(3):e70371. doi: 10.1002/cre2.70371 (PMC13180440; doi:10.1002/cre2.70371)
Supplement: Supplementary file 1 — Supporting File 1 [file CRE2-12-e70371-s002.docx]

Supplementary Table 1. Adjusted odds ratios (95% CI) for poorer glycemic control (HbA1c ≥ 7.0%) according to number of remaining teeth by sex

| **Remaining Teeth (n)** | Total population | | | Sex | | | | | | |
| --- | --- | --- | --- | --- | --- | --- | --- | --- | --- | --- |
|  |  |  |  | Men | | | Women | | | p for interaction |
|  | Unweighted n | Event n | Adjusted OR^*^  (95% C.I) | Unweighted n | Event n | Adjusted OR^*^  (95% C.I) | Unweighted n | Event n | Adjusted OR^*^  (95% C.I) |  |
| per 1 tooth (continuous) | 2146 | 1003 | **0.982  (0.967, 0.998)** | 1125 | 522 | 1.006  (0.983, 1.029) | 1021 | 481 | **0.956  (0.934, 0.979)** | **0.002** |
| p-value |  |  | **0.026** |  |  | 0.631 |  |  | **<0.001** |  |
| 0-19 | 615 | 278 | 1.370  (0.985, 1.905) | 318 | 141 | 0.853  (0.545, 1.336) | 297 | 151 | **2.400  (1.489, 3.869)** | **0.007** |
| 20-27 | 1121 | 468 | 1.213  (0.891, 1.652) | 598 | 280 | 0.933  (0.613, 1.419) | 523 | 249 | **1.634  (1.060, 2.519)** |  |
| ≥28 | 410 | 257 | 1 (Ref.) | 209 | 101 | 1 (Ref.) | 201 | 81 | 1 (Ref.) |  |
| p-value |  |  | 0.171 |  |  | 0.782 |  |  | **0.004** |  |

The p-values were obtained using logistic regression.
This model was adjusted for age, sex, body mass index, smoking status, alcohol consumption, regular exercise, education level, household income, and presence of hypertension, dyslipidemia and periodontitis.

*Abbreviation: OR, odds ratio.

Supplementary Table 2. Adjusted odds ratios (95% CI) for poorer glycemic control (HbA1c ≥ 7.0%) according to number of remaining teeth by sex and age group

| **Remaining Teeth (n)** | Men | | | | | |
| --- | --- | --- | --- | --- | --- | --- |
|  | Age 40-59 years | | | Age ≥60 years | | |
|  | Unweighted n | Event n | Adjusted OR (95% C.I) | Unweighted n | Event n | Adjusted OR (95% C.I) |
| per 1 tooth (continuous) | 421 | 227 | 0.992 (0.944, 1.043) | 704 | 295 | 1.009 (0.982, 1.037) |
| p-value |  |  | 0.755 |  |  | 0.509 |
| 0-19 | 54 | 28 | 0.995 (0.467, 2.117) | 264 | 113 | 0.771 (0.400, 1.483) |
| 20-27 | 231 | 130 | 0.976 (0.555, 1.716) | 367 | 150 | 0.894 (0.471, 1.695) |
| ≥28 | 136 | 69 | 1 (Ref.) | 73 | 32 | 1 (Ref.) |
| p-value |  |  | 0.996 |  |  | 0.664 |
| **Remaining Teeth (n)** | Women | | | | | |
|  | Age 40-59 years | | | Age ≥60 years | | |
|  | Unweighted n | Event n | Adjusted OR (95% C.I) | Unweighted n | Event n | Adjusted OR (95% C.I) |
| per 1 tooth (continuous) | 303 | 158 | 0.938 (0.855, 1.030) | 718 | 323 | **0.957 (0.933, 0.981)** |
| p-value |  |  | 0.179 |  |  | **<0.001** |
| 0-19 | 22 | 15 | 2.073 (0.607, 7.085) | 275 | 136 | **2.498 (1.321, 4.723)** |
| 20-27 | 161 | 87 | 1.589 (0.879, 2.872) | 362 | 162 | 1.697 (0.913, 3.155) |
| ≥28 | 120 | 56 | 1 (Ref.) | 81 | 25 | 1 (Ref.) |
| p-value |  |  | 0.395 |  |  | **0.008** |

The p-values were obtained using a logistic regression.

This model was adjusted for age, sex, body mass index, smoking status, alcohol consumption, regular exercise, education level, household income, and presence of hypertension and dyslipidemia.

*Abbreviation: OR, odds ratio.

Supplementary Table 3. Prevalence of periodontitis across different diabetes control groups.

| Groups | Periodontitis, % (SE) |
| --- | --- |
| HbA1c <6.5% | 52.89 (2.60) |
| HbA1c 6.5-6.9% | 48.09 (2.91) |
| HbA1c ≥7 | 56.23 (2.12) |
| p-value | 0.064 |
