# Supplementary material for: Tooth Loss and Uncontrolled Diabetes Among Korean Adults With Type 2 Diabetes: Insights From the Korea National Health and Nutrition Examination Survey (KNHANES) 2014–2018
Source: Clin Exp Dent Res. 2026 May 17;12(3):e70371. doi: 10.1002/cre2.70371 (PMC13180440; doi:10.1002/cre2.70371)
Supplement: Supplementary file 2 — Supporting File 2 [file CRE2-12-e70371-s001.docx]

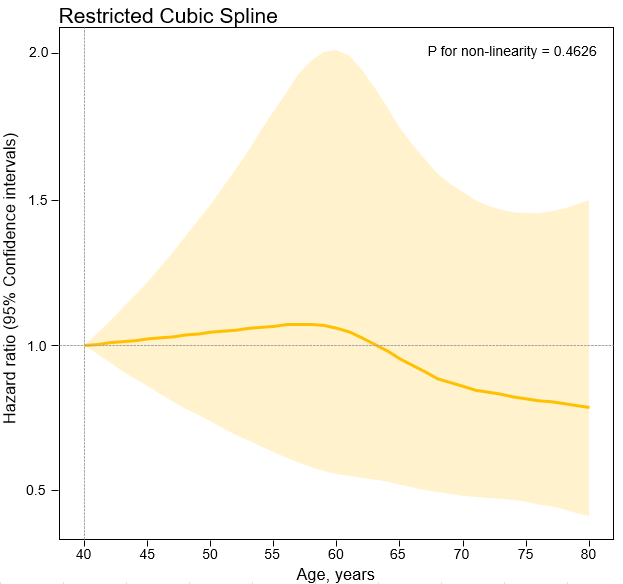


Supplementary Figure 1. Restricted Cubic Spline curve analysis was performed to analyzed whether any significant non-linearity existed among age and diabetes control. There was no significant non-linearity among age and diabetes control (p for non-linearity=0.4626), therefore, age was modeled as a linear variable.
